# Fast and Reliable Determination of Phthalic Acid Esters in the Blood of Marine Turtles by Means of Solid Phase Extraction Coupled with Gas Chromatography-Ion Trap/Mass Spectrometry

**DOI:** 10.3390/toxics9110279

**Published:** 2021-10-22

**Authors:** Ivan Notardonato, Cristina Di Fiore, Alessia Iannone, Mario Vincenzo Russo, Monica Francesca Blasi, Gabriele Favero, Daniela Mattei, Carmela Protano, Matteo Vitali, Pasquale Avino

**Affiliations:** 1 Department of Agriculture, Environmental and Food Sciences, University of Molise, I-86100 Campobasso, Italy; ivan.notardonato@unimol.it (I.N.); c.difiore@studenti.unimol.it (C.D.F.); a.iannone2@studenti.unimol.it (A.I.); mvrusso@unimol.it (M.V.R.); 2 Department of Chemistry and Technologies of Drug, University of Rome “La Sapienza”, I-00185 Rome, Italy; blasimf@yahoo.com (M.F.B.); gabriele.favero@uniroma1.it (G.F.); 3 Department of Environment and Health, Italian National Health Institute, I-00161 Rome, Italy; daniela.mattei@iss.it; 4 Filicudi Wildlife Conservation, Stimpagnato Filicudi, I-98050 Lipari, Italy; 5 Department of Public Health and Infectious Diseases, University of Rome La Sapienza, I-00185 Rome, Italy; carmela.protano@uniroma1.it (C.P.); matteo.vitali@uniroma1.it (M.V.)

**Keywords:** PAEs, blood, marine turtles, *Caretta caretta*, pollution, SPE, GC-IT/MS

## Abstract

The presence of phthalic acid esters (PAEs) in marine environments is an important issue. These chemicals are able to affect marine organisms, particularly marine turtles, and to act as endocrine disrupters. In this paper, for the first time, a simple and reproducible analytical method based on solid-phase extraction (SPE) coupled with gas chromatography—ion trap/mass spectrometry (GC-IT/MS) was developed for the extraction of phthalates from the blood of marine turtles. The extraction was obtained by using C_18_ phthalates-free as the stationary phase. In order to individuate the best working conditions for the extraction, the adsorption isotherms and breakthrough curves were studied. The overall analytical methodology was validated in terms of limit of detection (LOD, 0.08–0.6 ng mL^−1^), limit of quantification (LOQ, 0.4–0.8 ng mL^−1^), and correlation coefficients (>0.9933). By using this procedure, percentage recoveries ranging from 89 to 103% were achieved. The precision parameters (intra-day and inter-day) were studied, and the obtained values were smaller than 12.5%. These data confirm the goodness of the proposed analytical methodology, which is applied to real samples.

## 1. Introduction

Currently, marine environmental pollution by phthalic acid esters (PAEs) is a crucial topic, which is garnering global scientific attention. PAEs are regarded as the most ubiquitous chemicals, used in several industrial sectors for enhancing the properties of polymers, such as flexibility, softness, and workability [1,2]. Generally, finished plastic products contain 20–40% (*w*/*w*) PAEs [3]; as these compounds are not chemically bound to plastic polymer, but simply incorporated into it, they can leach from plastic and become widespread in the environment [4]. In this way, they may affect marine organisms, acting as endocrine disrupters (EDCs), and competing with the synthesis of endogenous hormones [5,6]. Several marine organisms are influenced by PAEs, such as plankton, macroalgae, piked dogfish (*Squalus acanthias*) [7], protozoans, invertebrates, mollusks, crustaceans, and different types of fishes [8]. Recently, PAEs were also detected in tissues of baleen whales [6] and different species of marine turtles in the Mediterranean Sea, an area particularly affected by marine plastic litter due to its slow turn over [9,10]. The most abundant marine turtle species in the Mediterranean Sea is the loggerhead sea turtle (*Caretta caretta*, Linnaeus, 1758), which is affected by the anthropogenic plastic debris that reach the sea every year [11]. Generally, this species feed on several prey species among invertebrates and vertebrates in both neritic and oceanic habitats, including those distributed along polluted coastal areas which can, in turn, ingest small plastic debris, accelerating the bioaccumulation of PAEs in marine turtles [12]. Ingestion of plastic particles also occurs since they confuse plastic residues for typical dietary products, by accidental ingestion when consuming natural prey, or from the direct uptake of chemicals from the surrounding seawater [13].

Given the habits and feeding patterns of *Caretta caretta*, they may be used as biomarkers for exposure to plastic by marine organisms, by analyzing chemicals derived from plastic in the blood [14]. For this purpose, an analytical protocol is necessary for extracting PAEs from an innovative matrix, such as the blood of marine turtles. Blood is chosen for two reasons: (i) given the delicate status of this species, in order to avoid animal stressing, this protocol could be considered a non-invasive sampling method [15]; and (ii), by determining the concentration of PAEs in the blood, it is possible to estimate the exposure of these organisms to these contaminants in live animals, and not only those which are stranded or deceased [16]. According to the data consulted, in fact, many studies on marine turtles were carried out only on turtles found stranded and deceased along Mediterranean coasts, which may significantly limit a correlation between the concentration of PAEs in biological tissues and other environmental parameters [9], and it may not accurately reflect the real health status of turtles [12]. To achieve these goals, for the first time, this paper aims to set up a protocol for extracting and determining PAEs in the blood of marine turtles. The procedure is based on solid-phase extraction (SPE) coupled with a gas chromatography–ion trap/mass spectrometry (GC-IT/MS). SPE was chosen as it allows for the extraction of a wide range of organic analytes from biological matrices (i.e., blood), achieving high extraction efficiencies. Moreover, SPE is more selective in comparison with liquid-liquid extraction (LLE) [17].

Blood is generally used for the evaluation of the health status of organisms (in particular, for humans) by means of analyzing its biochemical parameters, but the normal range of these parameters for turtles is not yet established, and cannot be used. In the literature, it is reported that xenobiotic substances such as polycyclic aromatic hydrocarbons (PAHs), heavy metals [18], and polychlorinated biphenyls (PCBs) [19] were determined in blood of turtles (Green Sea Turtle, *C. mydas* and Hawksbill Sea Turtle, *E. imbricata*), using these substances as health biomarkers for these marine organisms. It is hypothesized that these xenobiotic substances might reduce the hatching success of turtles, and determine the onset of new pathologies [12]. Furthermore, since the transport of exogenous substances is realized by means of blood, this matrix may be useful for monitoring chemicals present in turtles. This matrix can thus be used for estimating the health status of marine turtles [20]. Therefore, the analysis of PAEs in blood of marine turtles of the *Caretta caretta* species may be a way to trace the fate of these xenobiotic substances both in marine organisms, by predicting the load in internal tissues, and in the environment in general [18,19].

In this paper, the authors would like to propose a protocol for determining six PAEs in blood of marine turtles. PAEs investigated in this study are reported in Table 1, namely dimethyl phthalate (DMP), diethyl phthalate (DEP), di-isobutyl phthalate (DiBP), di-*n*-butyl phthalate (DBP), di-2-(ethylhexyl) phthalate (DEHP) and di-*n*-octyl phthalate (DnOP), whereas Table S1 of the Supplementary Material shows the relative chemical structures. The authors would like to underline that, after a thorough search of the literature, indeed this work is the first study for the detection of phthalates on blood of marine turtles. It should be noted that in this paper the authors chose to consider the di-isobutyl phthalate (DiBP) instead of benzyl butyl phthalate (BBP) due to its important and significant presence in several areas. Furthermore, DiBP is particularly spread in sea water. Since this work focuses on the research of PAEs in the most widespread marine turtle species, the authors tried to verify if DiBP is present at significant concentrations in *Caretta caretta* turtles living in the Mediterranean Sea. This could lead us to understand if this PAE is quite diffused, or not, also in the Mediterranean area [21].

## 2. Materials and Methods

### 2.1. Materials and Apparatus

**Figure 1:**
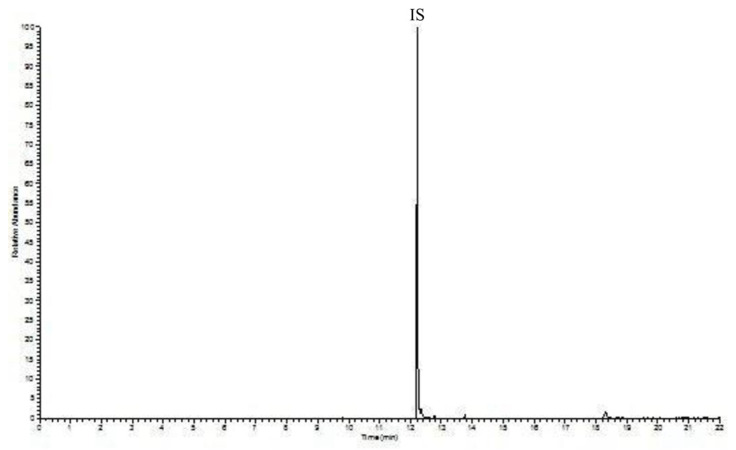
Blank chromatogram evidencing the absence of any PAE contamination in the lab glassware/reagents. The graph reported the relative abundance (*y* axis) as function of the time in min (*x* axis). For experimental conditions: see text.

In this study, DMP, DEP, DiBP, DBP, DEHP and DnOP were investigated in the blood of loggerhead sea turtles. Standards of phthalates were obtained from Sigma-Aldrich (Milan, Italy). Solvents used for the cleaning up and samples extraction such as acetone, *n*-heptane, methanol, methylene chloride, and cyclohexane, were obtained from Merks (Darmstadt, Germany). Phosphoric acid (85%) was used for the analysis. Phenanthrene was used as Internal Standard (IS), which was also obtained from Merks (Darmstadt, Germany). A solution of the investigated phthalates and the IS were prepared in acetone, with a concentration respectively equal to 500 ng mL^−1^ and 80 ng mL^−1^. Moreover, a solution of phosphoric acid/saline solution (9 g L^−1^) (1 + 1, *v*/*v*) (Darmstadt, Germany), necessary for the analysis, was prepared. In addition, it was possible for cross-contamination to occur during the sample collection and storage, as well as during the analytical stages, due to the solvents and environment, thus artificially increasing the measured concentrations [22]. In this study, to prevent cross-contamination, a severe cleaning procedure, deeply described in a previous paper [23], was applied; Figure 1 shows the blank chromatogram, evidencing the absence of any PAE contamination.

In this study, PAEs were analyzed by means of a Gas Chromatography—Ion Trap Mass Spectrometry (GC-IT/MS) analysis. A gas chromatograph (Thermo Fischer Scientific, Milan, Italy) model Finnigan TraceGC ULTRA equipped with a mass selective detector model (PolarisQ) and an analysis software (Xcalibur) was used. For the analysis, a fused-silica capillary column with a chemically bonded phase (SE–54.5% phenyl–95% dimethylpolysiloxane) from Teknokroma was used. The main characteristics were as follows: 30 m × 250 µm i.d.; d_f_ film thickness, 0.25 µm; theoretical plate number, *N*, 120,000 for *n*-dodecane at 90 °C; capacity factor, K_ı_, 7.3; optimum linear velocity of carrier gas, hydrogen, *u_opt_*, 34.5 cm s^−1^; utilization of theoretical efficiency, 95% was used [24,25]. The cartridges used in this study were 100 mg C_18_ phthalate-free (Chromabond).

### 2.2. Sampling

Loggerhead turtles investigated (*n* = 4) were rescued with detectable diseases or problems in the Aeolian Archipelago waters of the Mediterranean Sea (Sicily, Italy) (Table 2). All of the turtles were hospitalized in the Filicudi Wildlife Conservation First Aid Center for sea turtles and subjected to a physical/clinical health analysis applying the standard procedures [26]. Furthermore, for each turtle, the curved carapace length (CCL), the curved carapace width (CCW), and weight were measured (Table 2).

Blood (2 mL) was collected from the cervical sinus at the First Aid Center. After clinical investigation the remaining blood (1 mL) was placed in heparinized microtubes S-Monovette^®^ (7.5 mL LH, code 01.1604.400, orange). All the samples were stored at −20 °C. For blank analysis blood from turtles bred in captivity at the zoological center in Naples was collected and treated in the same way.

### 2.3. GC-IT/MS Conditions

For the gas chromatography analysis, helium was used as gas carrier at linear and constant velocity (ū of 34.5 cm s^−1^). 1 µL of sample was injected into the programmed temperature vaporizer (PTV) injector in splitless mode. Five seconds after the injection, the vaporizer was heated from 110–280 °C at 800 °C min^−1^, and kept constant for 5 min. After 2 min, the splitter valve was opened. The oven temperature was then programmed from 100–280 °C at 10 °C min^−1^ after injecting. The transfer line temperature was 270 °C. SCAN (electronic impact, EI, at 70 eV, mass range from 45–500 amu) and SIM (Selected Ion Monitoring) were used for the acquisition of data.

### 2.4. PAE Extraction of Blood from Loggerhead Sea Turtles (Caretta caretta)

Prior to use, the phthalate-free C_18_ cartridges were properly conditioned with 3 mL each of 3 organic solvents with different polarities (acetone, methanol, and *n*-heptane), and rinsed with distilled water. 1 mL of blood was diluted to 10 mL with a phosphoric acid/saline solution (1 + 1, *v*/*v*). The solutions obtained were passed through the C_18_ cartridge phthalates-free at a rate of approximately 5–6 mL min^−1^; the PAEs were then adsorbed by the stationary phase. Before the extraction by using solvent, the cartridge was dried with a gentle airflow for approximately 10 min. The analytes were desorbed from the cartridge with 1 mL of the methylene chloride by gravity flow. The eluate was collected in glass vials, and dried under a gentle nitrogen flow. The analytes were recovered with 200 µL of cyclohexane. 1µL was injected into the separation system for the analysis.

### 2.5. Adsorption Isotherms

For the adsorption isotherms, five solutions at different concentrations of PAEs were prepared in the range 50–200 ng mL^−1^. For the preparation of each solution, 1 mL of blood was diluted to 100 mL with the working solution (phosphoric acid/physiological solution, 1 + 1, *v*/*v*) in order to obtain the PAEs/C_18_ bonded-phase adsorption isotherms at 25 °C. For comparison, adsorbents (100 mg) were added to the working solutions (100 mL) and gently agitated for 24 h. The obtained suspension was then filtered, and the filtrate was extracted by means of the liquid-liquid extraction technique. The extracts were concentrated under a nitrogen flow. A sample was analyzed by GC-IT/MS.

### 2.6. Breakthrough Curves

In order to individuate the optimum working conditions, breakthrough curves were plotted for PAEs in the working solution on the C_18_ cartridges used. 1 mL of blood was diluted to 100 mL with the working solution. PAEs were subsequently extracted from 10 mL of the solution previously obtained by means of liquid-liquid extraction technique. The remaining 90 mL of the solution were passed through the C_18_ cartridge (100 mg) and collected in aliquots of 10 mL. The liquid-liquid extraction technique was then applied to all of the aliquots obtained in order to extract the analytes.

By analyzing the breakthrough curves, the chosen breakthrough volume was 20 mL, which will be explained later. The cartridges, previously conditioned, were thus enriched with 20 mL of the solution containing blood from turtles and the phosphoric acid/physiological solution (1 + 1, *v*/*v*). The samples, obtained from the working solution, were then shaken for approximately 2–3 min. After shaking, they were transferred into a glass vessel, which was connected to the C_18_ cartridge. The flow rate (5–6 mL min^−1^) was regulated and controlled by means of a vacuum pump (Vacuubrand, Westheim, Germany).

### 2.7. Study of the Extraction Solvent

An important factor pursued in this study was the choice of the best extraction solvent of PAEs from the C_18_ cartridge. The cartridges were enriched with 20 mL of the working solution spiked with 50 ppb of PAEs, and several solvents were tested: *n*-heptane, acetone, methanol, and methylene chloride. Before each extraction using the investigated solvent, the C_18_ cartridge was air-dried for approximately 5 min in order to remove the residual water. The adsorbed PAEs were then eluted by using organic solvent at approximately 2–3 mL min^−1^ through the cartridge.

**Figure 2:**
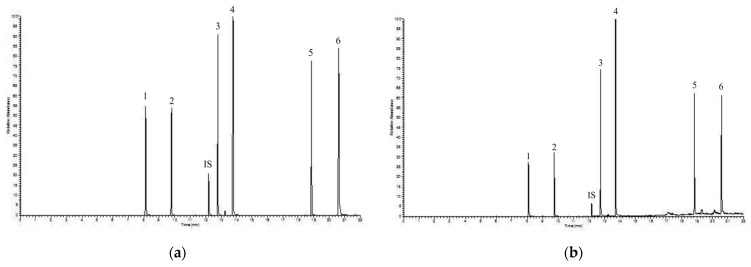
GC-IT/MS chromatograms of (**a**) PAEs standard solution, and (**b**) turtle blood sample spiked with a PAE solution at 75 ng mL^−1^. The graph reported the relative abundance (y axis) as a function of the time in min (*x* axis). Peaks: 1—DMP; 2—DEP; 3—DiBP; 4—DBP; 5—DEHP; 6—DnOP; IS (Internal Standard)—phenanthrene. For acronyms: see Table 1. For experimental conditions: see text.

In order to validate the extracting solvent, the operation was carried out by adding to the working solution two different concentrations of PAEs, respectively 10 ng mL^−1^ and 100 ng mL^−1^. In both cases, the recoveries were in line with the recoveries of the working solution spiked with 50 ng mL^−1^ concentration of PAEs. Figure 2 shows the GC-IT/MS chromatograms of both (a) the PAEs standard solution, and (b) that obtained by blood samples spiked with 75 ng mL^−1^.

### 2.8. Calibration Graphs

The calibration curves were obtained by plotting the ratio Area of the peak of each phthalate/Area of Internal Standard vs. concentration. For the construction of the calibration curves, solutions of known and increasing concentration were prepared. In this study 5 points were chosen for the construction of the calibration curves from 10–100 ng mL^−1^. All solutions were prepared from the same starting solution (500 ng mL^−1^). In each of the solutions the internal standard (4 µL) was added. 1 µL of each solution was injected into the CG-IT/MS.

### 2.9. Analysis of PAEs in Blood Samples

Before performing the SPE procedure, 1 mL of blood was spiked with 50 ppb of PAEs. This solution was then diluted to 10 mL with the working solution. The PAEs were extracted from the initial working solution by means of a liquid-liquid extraction technique proposed by Eckert et al. [27]. This step was essential for confirming the presence of phthalates in the initial solution.

An aliquot of 1 mL of blood was diluted to a 100 mL with a solution of aqueous phosphoric acid/physiological solution (1 + 1, *v*/*v*), containing PAEs and the Internal Standard (IS). This solution was extracted thrice with 10 mL of *n*-heptane. The apolar phase was then collected and placed inside a glass container. Subsequently, the solution was dried under a gentle nitrogen flow and recovered with 250 µL of methanol. Finally, 1 µL of this solution was injected into the separation system.

## 3. Results and Discussion

### 3.1. Evaluation of the Analytical Methodology

The main purpose of this paper was to develop a method for the extraction of plasticizers (i.e., PAEs) from the blood of marine turtles. For this scope, analytical parameters were determined, such as the adsorption isotherms, breakthrough curves, and the best extraction solvent.

**Figure 3:**
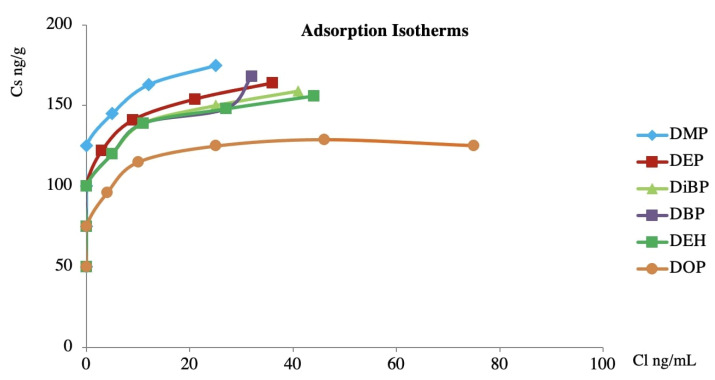
Adsorption isotherm curves at 25 °C of PAEs between the C_18_ adsorbent and working solution.

The first step was the study of the adsorption isotherms for PAEs and the stationary phase (C_18_), reported in Figure 3.

The isotherm curves showed that the stationary phase (C_18_) was able to significantly adsorb the PAEs at a low concentration, whereas high concentrations were less adsorbed. Furthermore, the lower molecular weight compounds showed a distribution in favor of the stationary phase, whereas the higher molecular weight compounds showed a distribution in favor of the stationary phase only when their concentration was lower. From the reported curves, it is possible to hypothesize that the molecular weight of the compounds influences their behavior.

**Figure 4:**
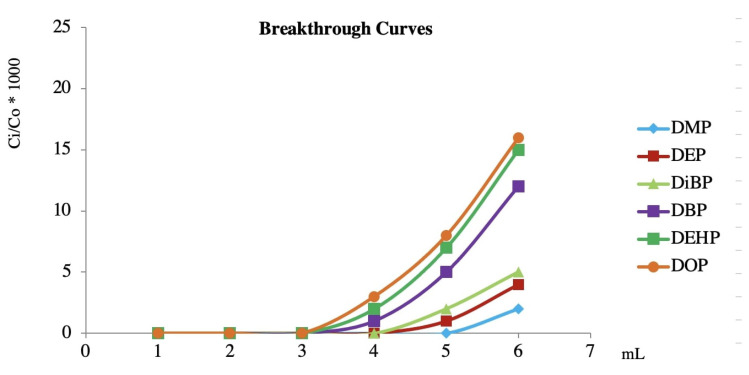
Breakthrough curves for the PAEs on the C_18_ cartridge (100 mg).

The second step was the study of the breakthrough curves. The breakthrough curves (C_i_/C_o_ × 100 vs. volume of blood diluted with the working solution) of PAEs are reported in Figure 4. C_i_/C_o_ is the ratio between the concentration of solute in effluent (C_i_) and the concentration of the incoming solute (C_o_). The theoretical breakthrough volume, extrapolated from the curves, was approximately 30 mL for all investigated compounds. Generally, 80% of the theoretical volume studied can be used as the experimental breakthrough volume. In addition, considering the importance of the matrix and the limited availability of loggerhead sea turtle blood, the chosen experimental volume was 20 mL, which could be used without any losses.

The choice of the best extraction solvent of PAEs from the C_18_ stationary phase was an incredibly important step in this protocol. The extraction solvent had to show high extraction efficiency for the target molecules. For this reason, four solvents were considered. The percentage recoveries obtained through use of the investigated solvents are shown in Table 3. All solvents were tested on 1 mL fractions for a total of 4 mL for each solvent.

The reported recoveries were obtained by using four different extraction solvents, characterized by different polarities. Acetone and methanol showed scarce recoveries of PAEs, respectively 22–57% and 25–52%. *n*-Heptane, by contrast, was able to provide acceptable recoveries, between 63 and 76%. The best recoveries were obtained by using methylene chloride, between 89 and 103%. For this reason, good recoveries were obtained by using an apolar solvent, whereas through the use of a polar solvent, the PAE recoveries were not considered analytically significant. Due to the scarcity of blood samples available, three replicates were carried out for the determination of the percentage recoveries only for the solvent which showed the highest recoveries.

### 3.2. GC-IT/MS Method Validation

For the validation of the analytical method, different analytical parameters, such as limit of detection (LOD), limit of quantification (LOQ), calibration data, percentage recoveries, and method reproducibility were studied; in particular, LODs and LOQs, ranging between 0.08–0.6 ng mL^−1^ and 0.4–0.8 ng mL^−1^, respectively, and calibration data, are showed in Table 4.

For each PAE investigated, the LODs and LOQs were determined according to Knoll’s definition [28]: they were appropriate for determining this group of plasticizers in blood samples. Regarding the calibration data, the linearity of the response for all of the PAEs analyzed was studied in the range of 1–500 ng mL^−1^ (*R*^2^ > 0.9933). Precision and accuracy of the analytical methodology were determined, investigating the intra- and inter-day errors. Inter-day data were determined by intra-day error propagation, and for each intra-day three reruns were carried out.

The results, reported in Table 5, showed that the proposed method allowed for the achievement of good percentage recoveries, in a range within 91.3–100.6%, with a relative standard deviation (RSD) smaller than 12.5%.

**Figure 5:**
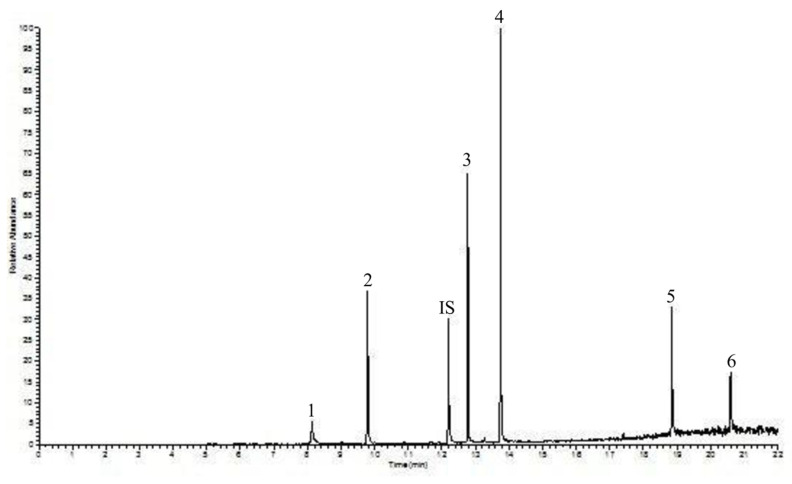
GC-IT/MS chromatogram of PAEs obtained by analyzing a real sample of blood from *Caretta caretta* turtle. The graph reported the relative abundance (*y* axis) as function of the time in min (*x* axis). Peaks: 1—DMP; 2—DEP; 3—DiBP; 4—DBP; 5—DEHP; 6—DnOP; IS (Internal Standard)—phenanthrene. For experimental conditions: see text.

Moreover, as previously mentioned, another goal pursued by the authors was to control cross-contamination, to avoid obtaining artificial results. Cross-contamination with phthalates was a crucial aspect in this study, since the aim of this work was to set up a new method for the extraction of PAEs from an innovative matrix. For this reason, all of the solvents used were subjected to a rigorous procedure, as reported in [23]. The result was a very neat chromatogram, as described above, where PAEs could be easily and successfully identified and determined without issue. Figure 5 shows the chromatogram of a turtle blood sample.

### 3.3. Similar Studies

A goal pursued in this paper was to set up a protocol for the extraction of PAEs from blood samples from marine turtles, based on SPE coupled with GC-IT/MS. The Scopus database does not report papers dealing with the determination of PAEs in the blood of marine turtles and of organisms in general (humans included), and this confirms the difficulty and issues in extracting this group of chemicals from a complex biological matrix. For this reason, the authors compared their results with those present in the scientific literature. In particular, the determination of PAHs in the blood of a marine turtle species (*C. mydas*) is reported. Sinaei et al. [12] determined 16 PAHs in the blood of green sea turtles (*C. mydas*) by means of liquid chromatography with fluorescence detection, and the only analytical parameter reported was the percentage recovery. The analytical methodology allowed the authors to obtain percentage recoveries higher than 85%. The levels of PAHs in that species of marine turtle were significant, ranging between 2.5 and 19%. The authors also compared their results in terms of LODs and LOQs with previously published literature. Ebrahim et al. 2017 [29] determined traces of PAEs in human plasma by means of the dispersive liquid-liquid microextraction, and a gas chromatography with mass spectrometry analysis. For all of the analytes, they obtained an LOD ranging between 1.06 and 1.60 ng mL^−1^, and values of LOQs ranging between 3.5 and 5.3 ng mL^−1^, which were higher than those obtained in this study.

It is possible to state that both methods are effective in terms of recoveries, allowing the determination of two different classes of contaminants in the blood of marine turtles. Furthermore, Ehsanpour et al. [18] determined heavy metals in the blood of several species of marine turtle by using atomic absorption spectrophotometry (AAS), demonstrating the contamination of marine turtles by heavy metals in significant concentrations. The authors underlined that their results were in line with those already present in the scientific literature.

### 3.4. Application to Real Samples

The overall method was applied to the blood of turtles rescued in the Mediterranean Sea. The determinations of investigated PAEs were carried out by means of GC-IT/MS, and the results are reported in Table 6.

By consulting the obtained data, it is possible to state that the proposed extraction method is reproducible, reliable, sensitive, and allows for a quantitative and qualitative determination of PAEs in the blood of marine turtles. By using the SPE, it was possible to carry out a clean-up of the investigated matrix, facilitating the determination of PAEs by means of the GC-IT/MS.

From the scientific literature, it is known that phthalates are one of a group of organic compounds which are present in aquatic organisms [30]. Different processes, such as leaching and atmospheric deposition, are the main sources of phthalates in aquatic systems [31]. The obtained data confirm the presence of phthalates in *Caretta caretta* turtles. Looking at the data, DBP is the most commonly found phthalate in *Caretta caretta* blood, with a concentration ranging between 6 and 57 ng mL^−1^, which already is on the list of priority compounds of the Convention for the Protection of the Marine Environment of the North-East Atlantic (OSPAR Convention) [32]. Moreover, DiBP, DEHP, and DnOP have been found in three samples of blood, with concentrations ranging between 19 and 41 ng mL^−1^, 40–53 ng mL^−1^, and 13–37 ng mL^−1^, respectively, whereas DMP and DEP have been found in one turtle with a concentration of between 14 and 74 ng mL^−1^, respectively. Considering these data, it is complex to identify information on the PAEs sources. Environmental conditions such as water salinity and UV radiation may affect the release of additives specifically for each analyte and each type of plastic material. In addition, studies suggested that areas with high levels of turbulence, such as coastlines, might determine a more efficient release of additives from all types of plastic. PVC is capable of leaching the major concentration of phthalates, particularly of DEHP, which is added during PVC production in order to make it soften [31]. For this reason, the significant concentration of DEHP in the blood of turtles may be due to its crucial leaching from PVC plastic litter present in the seawater. In addition, the ability of DEHP to partially solubilize in biological fluids, such as blood and blood plasma, has been demonstrated [33]. DiBP was found in three samples at concentrations ranging between 19 and 41 ng mL^−1^, showing the presence of this PAE in the blood of *Caretta caretta*. DiBP was, in fact, detected in several areas, and its own presence was particularly important in sea water [21]. Regarding DBP, it is heavily used during the plastic material production, since it is able to add flexibility to plastic. DBP has been found in all analyzed blood samples; similar results are shown in the scientific literature, where the prevalence of DBP and DEHP in *Caretta caretta* tissues is reported [9]. In one blood sample, only DBP and DEP were found in only one turtle, with a concentration of 6 ng mL^−1^ and 74 ng mL^−1^, respectively. This could be due to a different diet of the turtle. Loggerhead sea turtles generally consume benthic invertebrates [34], which may ingest hydrophobic contaminants such as PAEs [35]. However, an important variability in terms of prey species in the Mediterranean Sea has been proven [34]. Therefore, the variability in terms of feeding habits may have influenced the concentration of PAEs found. However, the different concentration of phthalates in the blood of the analyzed turtles did not show a significant correlation with the biometric parameters [9]. Furthermore, no information was available regarding the history of the marine animal (e.g., age, area in which it lived, etc.). Considering the potential risk associated with PAEs, a risk assessment is required. The ecological risks deriving from these widespread chemicals impact various marine species. More sensitive fish species are affected by the presence of DiBP, whereas DBP is less dangerous for sensitive fish species. The most important ecological risk derives from the presence of DEHP in the seas, which is capable of determining a high risk for all marine organisms. Since this group of compounds is considered a threat for the health status of marine organisms and the sea in general, further studies will be conducted for an ecological risk assessment in the Mediterranean Sea [36,37].

## 4. Conclusions

The most important aim pursued in this study was to set up a protocol for the ex-traction of PAEs in a very sensitive matrix, i.e., blood. Even if blood is a well investigated matrix, an analytical procedure for analyzing PAEs in this matrix is not yet present, due to difficulties related both to the biological matrix and to the compounds, which causes difficulties in its analysis. The extracting protocol proposed by the authors would be optimal for a sensitive and reproducible analysis of PAEs in the blood of marine turtles. The protocol allowed for the determination of seven widespread PEAs with reduced analysis times and in the absence of a matrix effect. By using SPE, the obtained LODs and LOQs were between 0.08–0.6 ng mL^−1^, and a recovery between 89 and 103% was achieved, which was in line with those reported in similar studies published in the scientific literature [38]. This method is therefore useful and interesting for a rapid and non-invasive determination of PAEs in live marine turtles, and is also an inexpensive procedure. By applying this procedure, the obtained results showed the presence of PAEs in the blood of marine turtles, with a concentration raging between 6 and 74 ng mL^−1^ of blood. In addition, the authors hypothesize that, by using this extracting protocol, it will be possible to carry out the extraction of these chemicals from the blood of other species of marine turtles (and other marine organisms) in order to implement the scientific literature.

## Figures and Tables

**Table 1:** Phthalates investigated in this paper, along with their corresponding abbreviations, formulae, molecular weight (MW), and target and qualifier ions.

Phthalate	Abbreviation	Formula	MW	Target Ion	Qualifier Ion
Dimethyl Phthalate	DMP	C_10_H_10_O_4_	194.18	163	194
Diethyl Phthalate	DEP	C_12_H_14_O_4_	222.24	149	177
Di-*iso*butyl Phthalate	DiBP	C_16_H_22_O_4_	278.34	149	205
*n*-Dibutyl Phthalate	DBP	C_16_H_22_O_4_	278.34	149	205
Bis-(2-Ethylhexyl) Phthalate	DEHP	C_24_H_38_O_4_	390.56	149	167
Di-*n*-octyl Phthalate	DnOP	C_24_H_38_O_4_	390.56	149	261

**Table 2:** Identification code of turtles investigated in this paper, along with their corresponding rescue areas, curved carapace length (CCL), curved carapace width (CCW), and weight.

Identification Code of Turtle	Rescue Area	CCL (cm)	CCW (cm)	Weight (kg)
A	Sicily	70.5	64.0	43.0
B	Sicily	54.0	50.0	20.0
C	Sicily	62.5	55.4	26.7
D	Sicily	69.0	63.0	42.0

**Table 3:** Mean recoveries (%), along with standard deviation of the seven investigated PAEs from the C_18_ cartridge by means of different organic solvents. For acronyms: see Table 1.

Solvent	Recovery (% ± s.d ^1^)					
	*DMP*	*DEP*	*DiBP*	*DBP*	*DEHP*	*DnOP*
Acetone	52.2 ± 5.3	57.0 ± 6.2	42.5 ± 4.6	31.3 ± 6.8	22.6 ± 8.9	32.4 ± 9.4
Methanol	45.6 ± 6.7	41.4 ± 7.3	28.4 ± 6.8	25.5 ± 8.4	37.0 ± 9.3	51.9 ± 8.8
*n*-Heptane	69.2 ± 4.2	76.3 ± 3.7	63.2 ± 5.1	67.2 ± 5.7	71.2 ± 4.8	63.9 ± 6.4
Methylene chloride	96.5 ± 4.9	97.8 ± 5.1	89.5 ± 9.2	103.1 ± 8.3	99.4 ± 7.4	92.2 ± 5.3

^1^ s.d.: standard deviation.

**Table 4:** Analytical parameters studied in the range 1–500 ng mL^−1^ by means of GC-IT/MS: correlation coefficients (R^2^), limit of detection (LOD), limit of quantification (LOQ), and percentage recoveries of spiked samples at two different concentrations for each PAE investigated in this study.

PAE	R^2^	LOD	LOQ	Recovery	Inter-Day
		(ng mL^−1^)	(ng mL^−1^)	Low conc. ^1^	High conc. ^2^
DMP	0.9989	0.6	0.8	91.4 ± 4.9	94.8 ± 5.1
DEP	0.9985	0.3	0.8	94.1 ± 6.0	92.5 ± 5.8
DiBP	0.9971	0.1	0.7	96.8 ± 7.3	99.5 ± 9.3
DBP	0.9965	0.08	0.7	99.7 ± 9.2	102.5 ± 7.4
DEHP	0.9933	0.08	0.4	96.3 ± 7.6	93.9 ± 8.1
DnOP	0.9958	0.1	0.4	93.1 ± 5.4	90.1 ± 6.2

^1^ Recoveries (mean % ± s.d.) obtained from real sample spiked with 10 ng mL^−1^ of PAE mixture solution; ^2^ recoveries (mean % ± s.d.) obtained from real sample spiked with 100 ng mL^−1^ of PAE mixture solution.

**Table 5:** Inter- and intra-day precision (%) with relative standard deviation (%).

PAE	Intra-Day 1	RSD ^1^ (%)	Intra-Day 2	RSD (%)	Inter-Day	RSD (%)
DMP	91.3 ± 3.1	3.4	95.7 ± 4.0	4.2	93.5 ± 5.1	5.5
DEP	98.8 ± 4.0	4.0	85.6 ± 3.4	4.0	92.2 ± 5.2	5.6
DiBP	100.6 ± 7.4	7.4	95.7 ± 8.3	8.7	98.2 ± 11.1	11.3
DBP	96.9 ± 8.3	8.6	104.3 ± 9.5	9.1	100.6 ± 12.6	12.5
DEHP	99.6 ± 5.2	5.2	90.1 ± 6.1	6.8	94.9 ± 8.0	8.4
DnOP	86.3 ± 3.6	4.2	96.3 ± 4.3	4.5	91.3 ± 5.6	6.1

^1^ RSD: relative standard deviation.

**Table 6:** PAEs concentration levels (ng mL^−1^) determined in the blood of *Caretta caretta* turtles. For turtle codes: see Table 2. For PAE acronyms: see Table 1.

Turtle Code	DMP	DEP	DiBP	DBP	DEHP	DnOP
A	<LOD	<LOD	<LOD	6	<LOD	<LOD
B	<LOD	<LOD	22	25	40	37
C	14	74	41	57	53	21
D	<LOD	<LOD	19	26	40	13

## Supplementary Materials

The following are available online at https://www.mdpi.com/article/10.3390/toxics9110279/s1, Table S1: Phthalates (PAEs) investigated in this paper, with their corresponding abbreviations, chemical structure, CAS number, chemical formula, molecular weight (MW) and Selected Ion Monitoring (SIM).
