# Supplementary material for: Fast and Reliable Determination of Phthalic Acid Esters in the Blood of Marine Turtles by Means of Solid Phase Extraction Coupled with Gas Chromatography-Ion Trap/Mass Spectrometry
Source: Toxics. 2021 Oct 22;9(11):279. doi: 10.3390/toxics9110279 (PMC8624151; doi:10.3390/toxics9110279)
Supplement: Supplementary file 1 [file toxics-09-00279-s001.zip › toxics-1397422-supplementary.pdf]

toxics MDPI

Supplementary Materials: Fast and Reliable Determination of Phthalic Acid Esters in the Blood of Marine Turtles by means of Solid Phase Extraction coupled with Gas Chromatography-Ion Trap/Mass Spectrometry

Ivan Notardonato, Cristina Di Fiore, Alessia Iannone, Mario Vincenzo Russo, Monica Francesca Blasi, Gabriele Favero, Daniela Mattei, Carmela Protano, Matteo Vitali and Pasquale Avino

Table S1. Phthalates (PAEs) investigated in this paper, with their corresponding abbreviations, chemical structure, CAS number, chemical formula, molecular weight (MW) and Selected Ion Monitoring (SIM).

Phthalate	Abbreviation	CAS number	Chemical structure	Formula	MW
Dimethyl phthalate	DMP	131-113	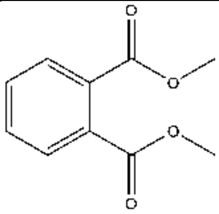	$C_{10}H_{10}O_4$	194.18
Diethyl phthalate	DEP	84-66-2	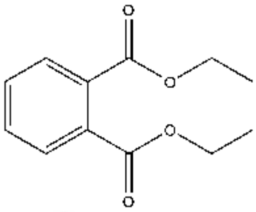	$C_{12}H_{14}O_4$	222.24
Di-*iso*butyl phthalate	DiBP	84-69-5	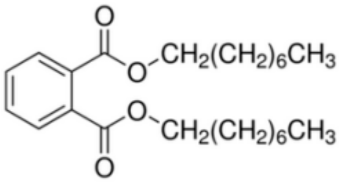	$C_{16}H_{22}O_4$	278.34
n-Dibutyl phthalate	DBP	84-74-2	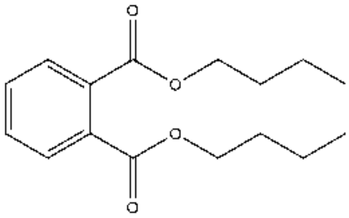	$C_{16}H_{22}O_4$	278.34
Bis-(2-ethylhexyl) phthalate	DEHP	117-81-7	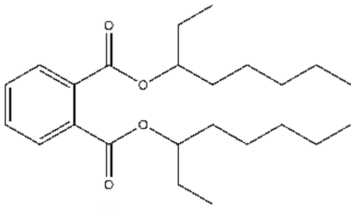	$C_{24}H_{38}O_4$	390.56
Di-octyl phthalate	DnOP	117-84-0	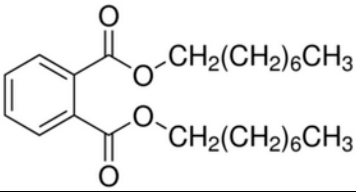	$C_{24}H_{38}O_4$	390.56
